# Supplementary figures and images for: Vehicle trajectory prediction and generation using LSTM models and GANs
Source: PLoS One. 2021 Jul 1;16(7):e0253868. doi: 10.1371/journal.pone.0253868 (PMC8248611; doi:10.1371/journal.pone.0253868)

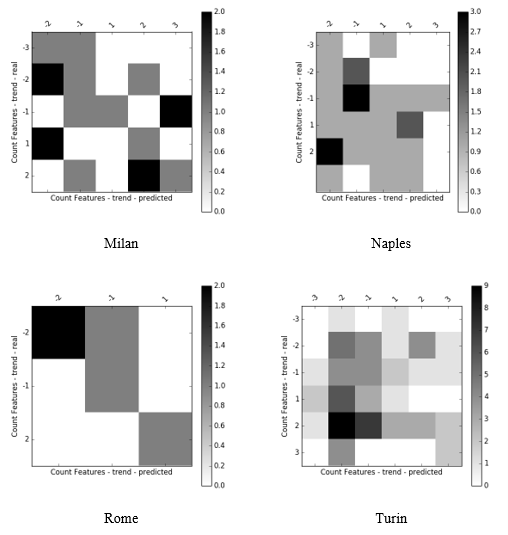

Supplement: S1 File — (ZIP) [file pone.0253868.s001.zip › images/ConfusionMatrices_100_CountTrend.png]

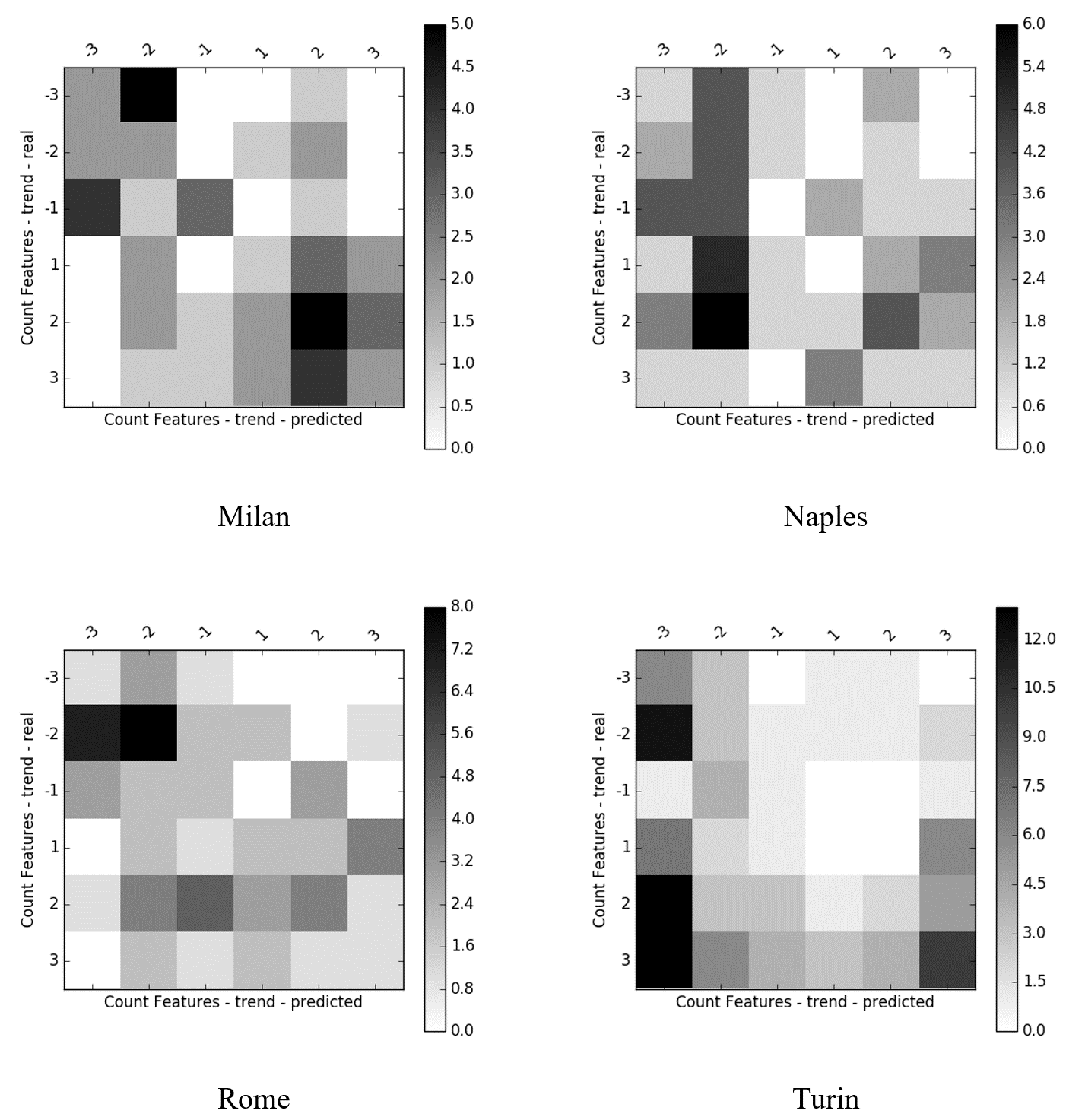

Supplement: S1 File — (ZIP) [file pone.0253868.s001.zip › images/ConfusionMatrices_CountTrend.png]

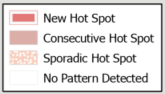

Supplement: S1 File — (ZIP) [file pone.0253868.s001.zip › images/HotSpot_legend.png]

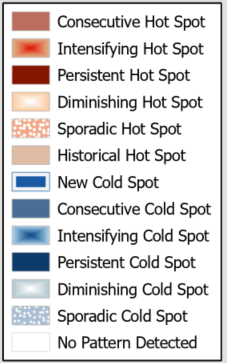

Supplement: S1 File — (ZIP) [file pone.0253868.s001.zip › images/HotSpot_legend_Milano.png]

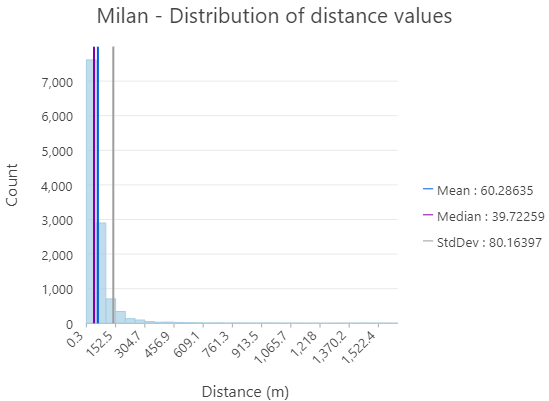

Supplement: S1 File — (ZIP) [file pone.0253868.s001.zip › images/Milano_NearTable_Dist.png]

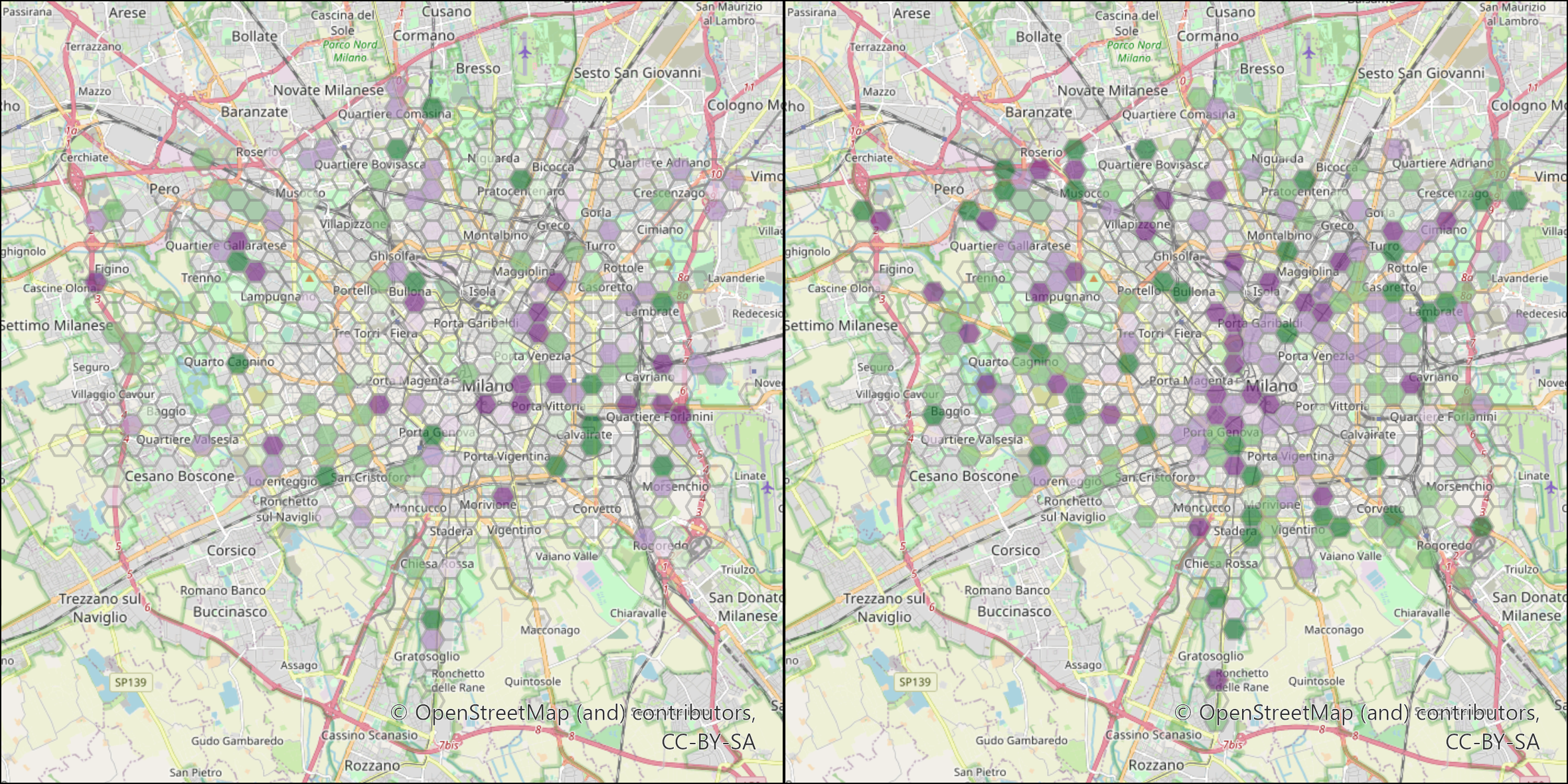

Supplement: S1 File — (ZIP) [file pone.0253868.s001.zip › images/Milano_real_vs_predicted_12 points_500m_fullExtent.png]

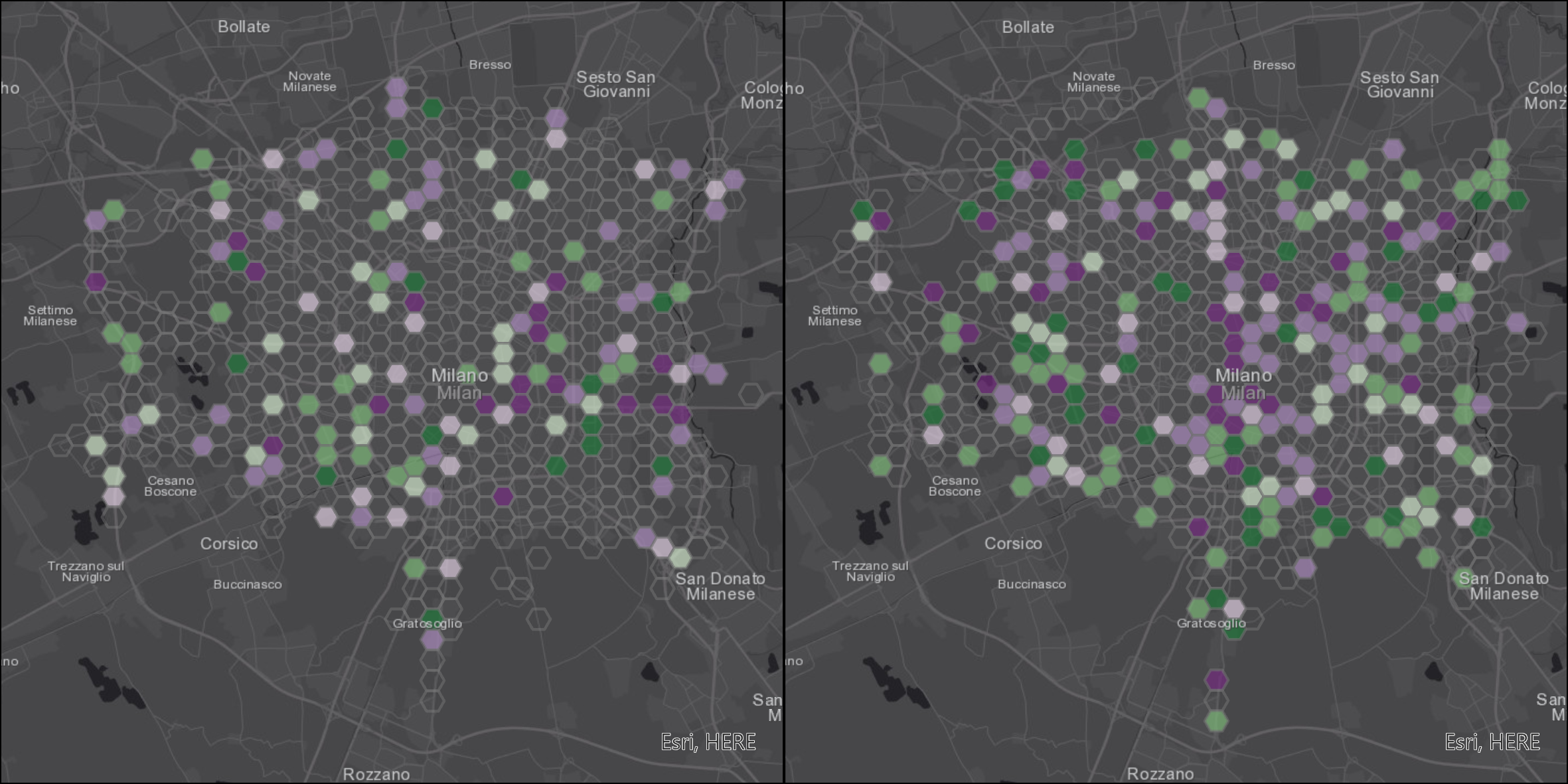

Supplement: S1 File — (ZIP) [file pone.0253868.s001.zip › images/Milano_real_vs_predicted_12 points_500m_fullExtent_gray.png]

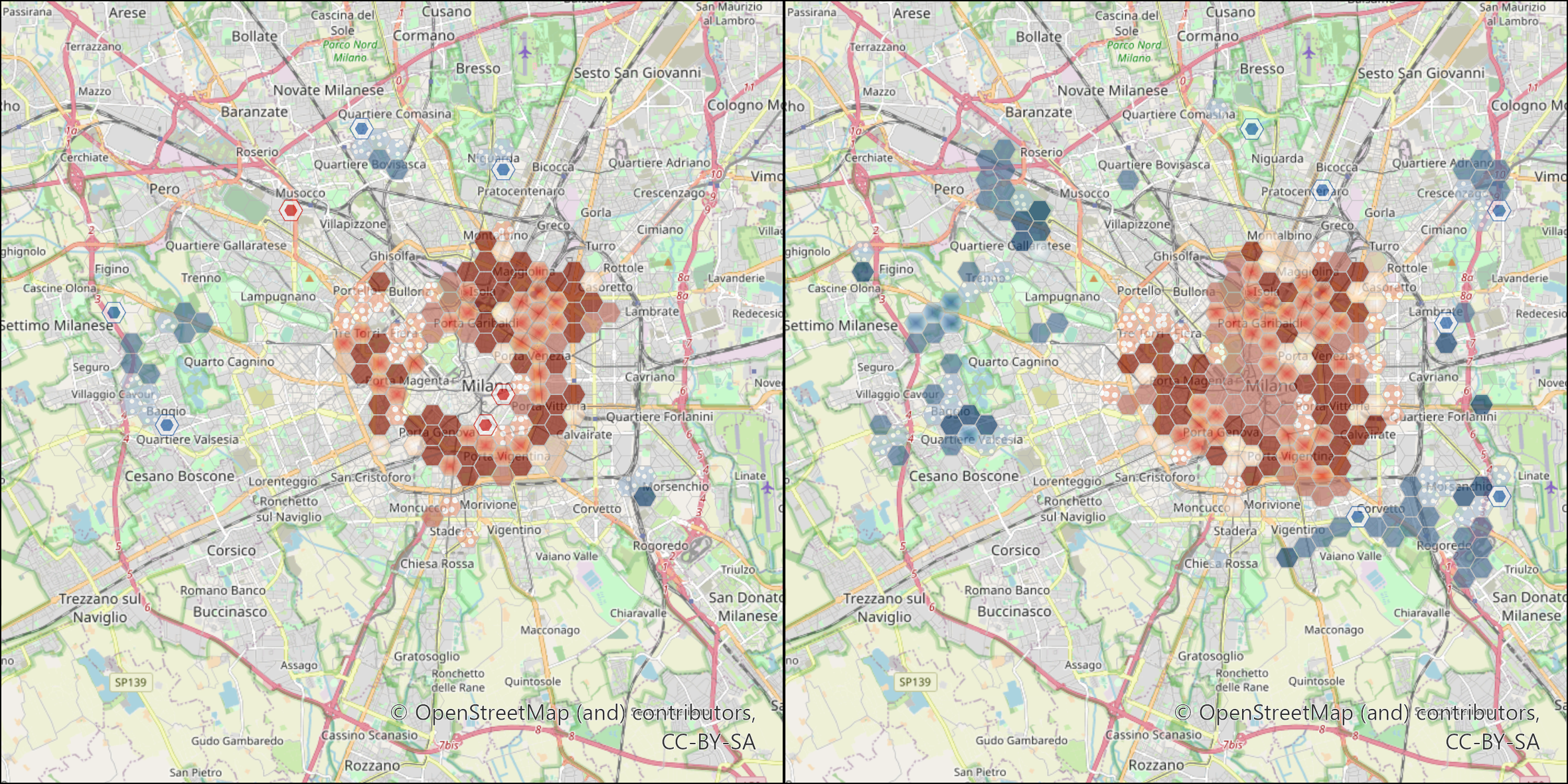

Supplement: S1 File — (ZIP) [file pone.0253868.s001.zip › images/Milano_real_vs_predicted_12 points_500m_HSA_fullExtent.png]

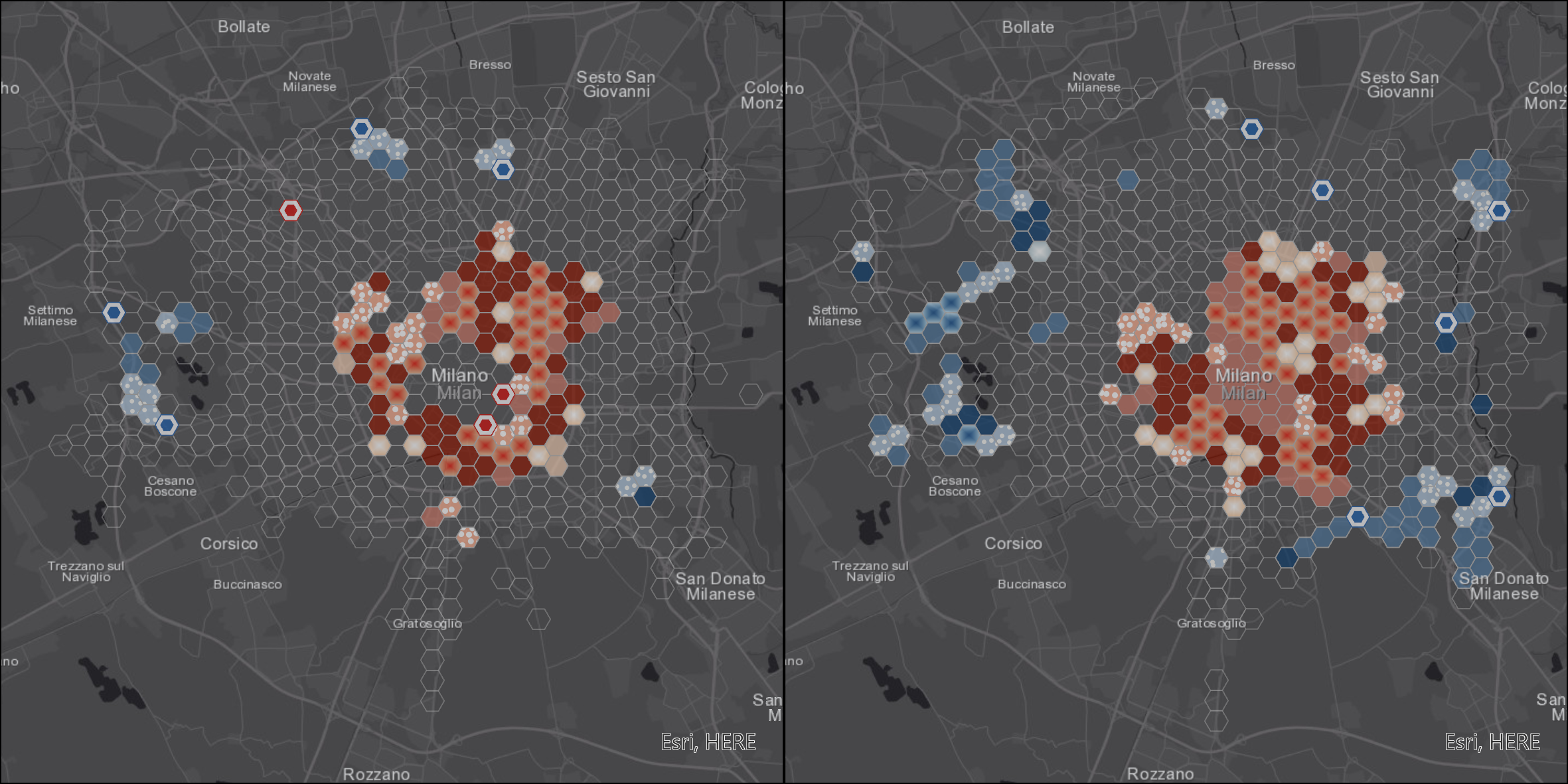

Supplement: S1 File — (ZIP) [file pone.0253868.s001.zip › images/Milano_real_vs_predicted_12 points_500m_HSA_fullExtent_gray.png]

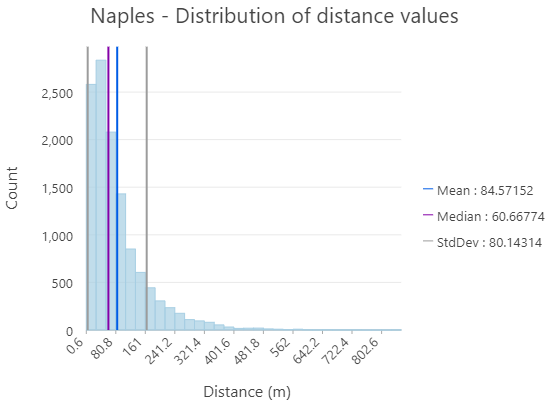

Supplement: S1 File — (ZIP) [file pone.0253868.s001.zip › images/Napoli_NearTable_Dist.png]

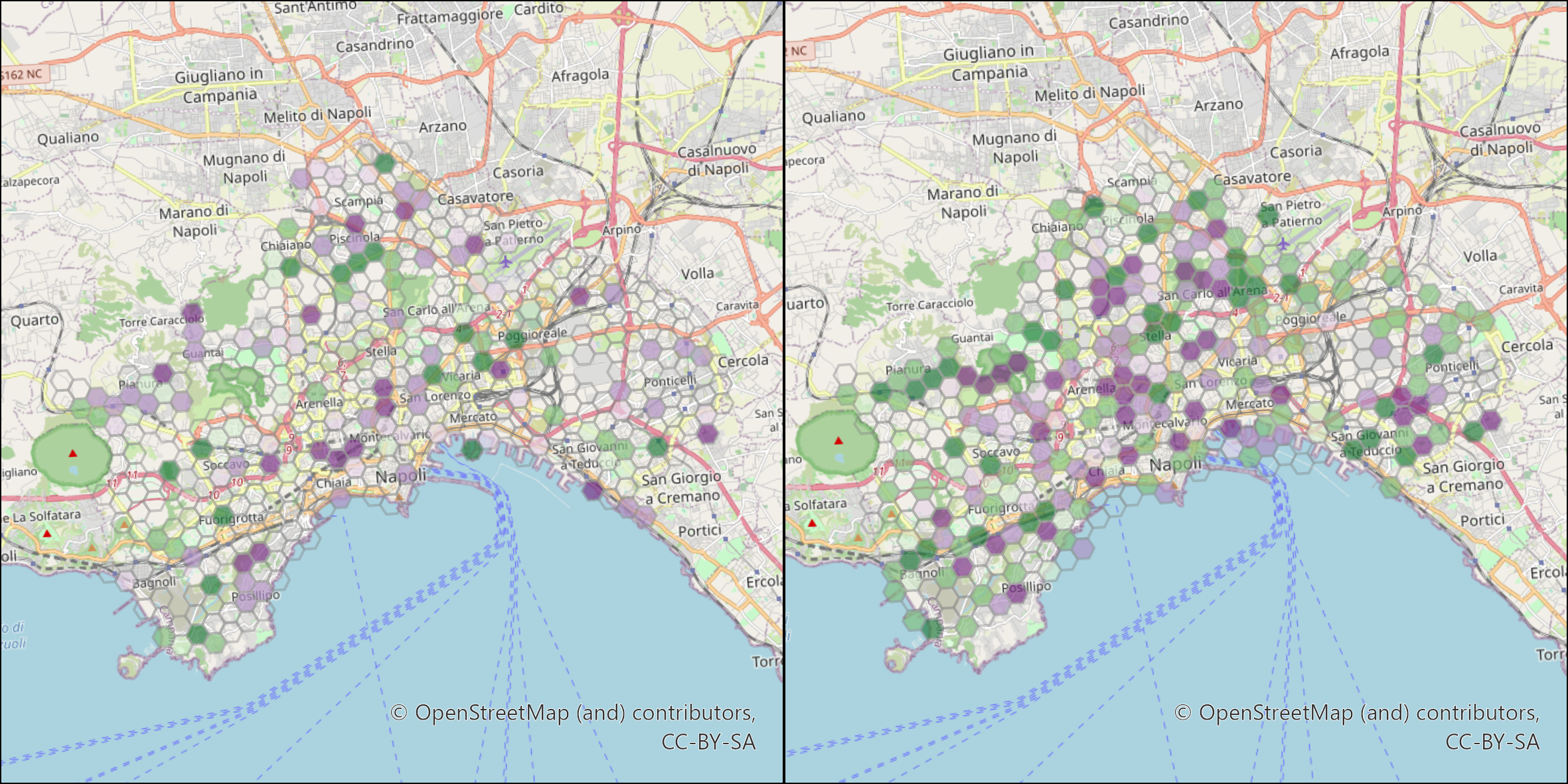

Supplement: S1 File — (ZIP) [file pone.0253868.s001.zip › images/Napoli_real_vs_predicted_12 points_500m_fullExtent.png]

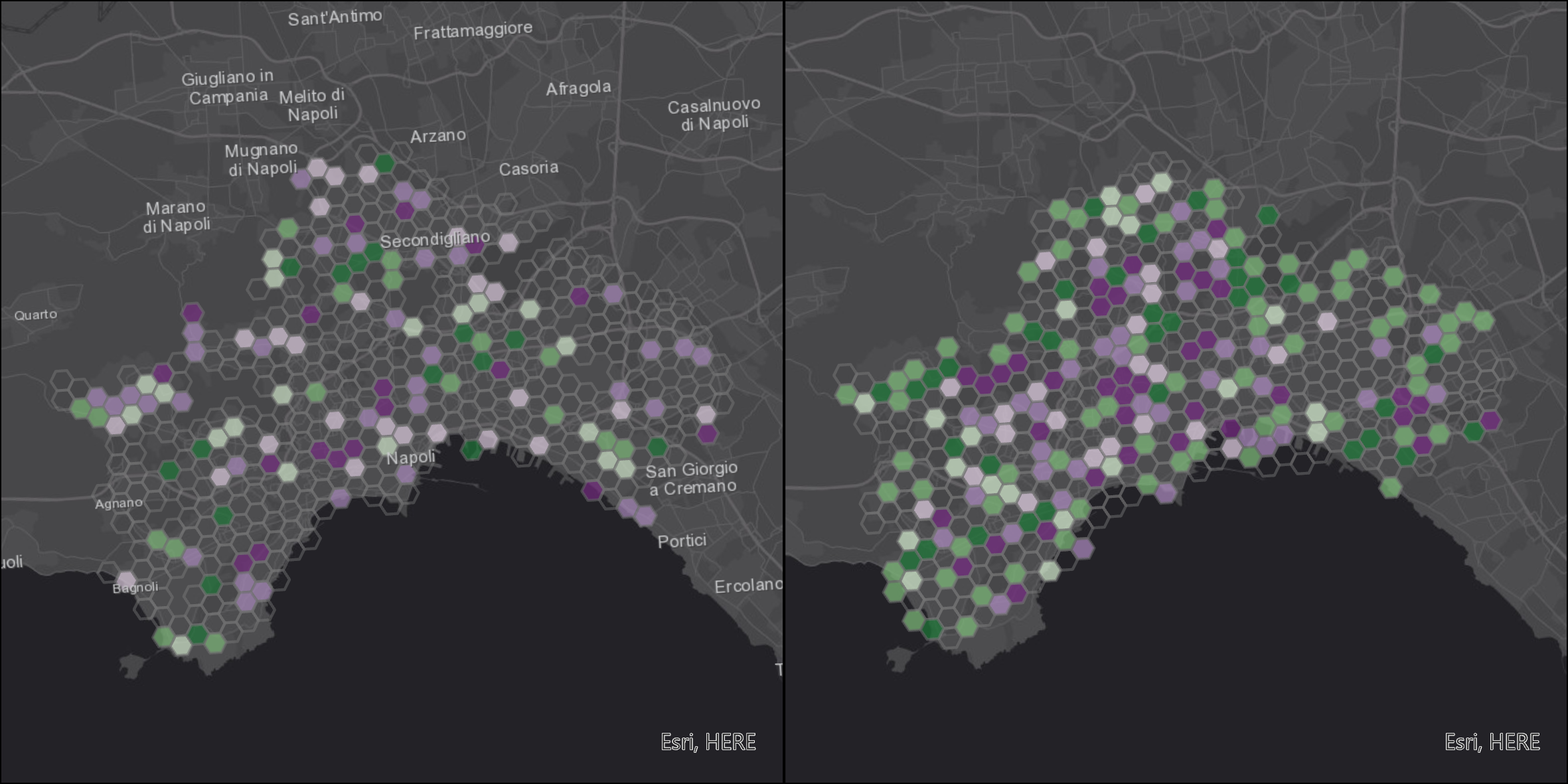

Supplement: S1 File — (ZIP) [file pone.0253868.s001.zip › images/Napoli_real_vs_predicted_12 points_500m_fullExtent_gray.png]

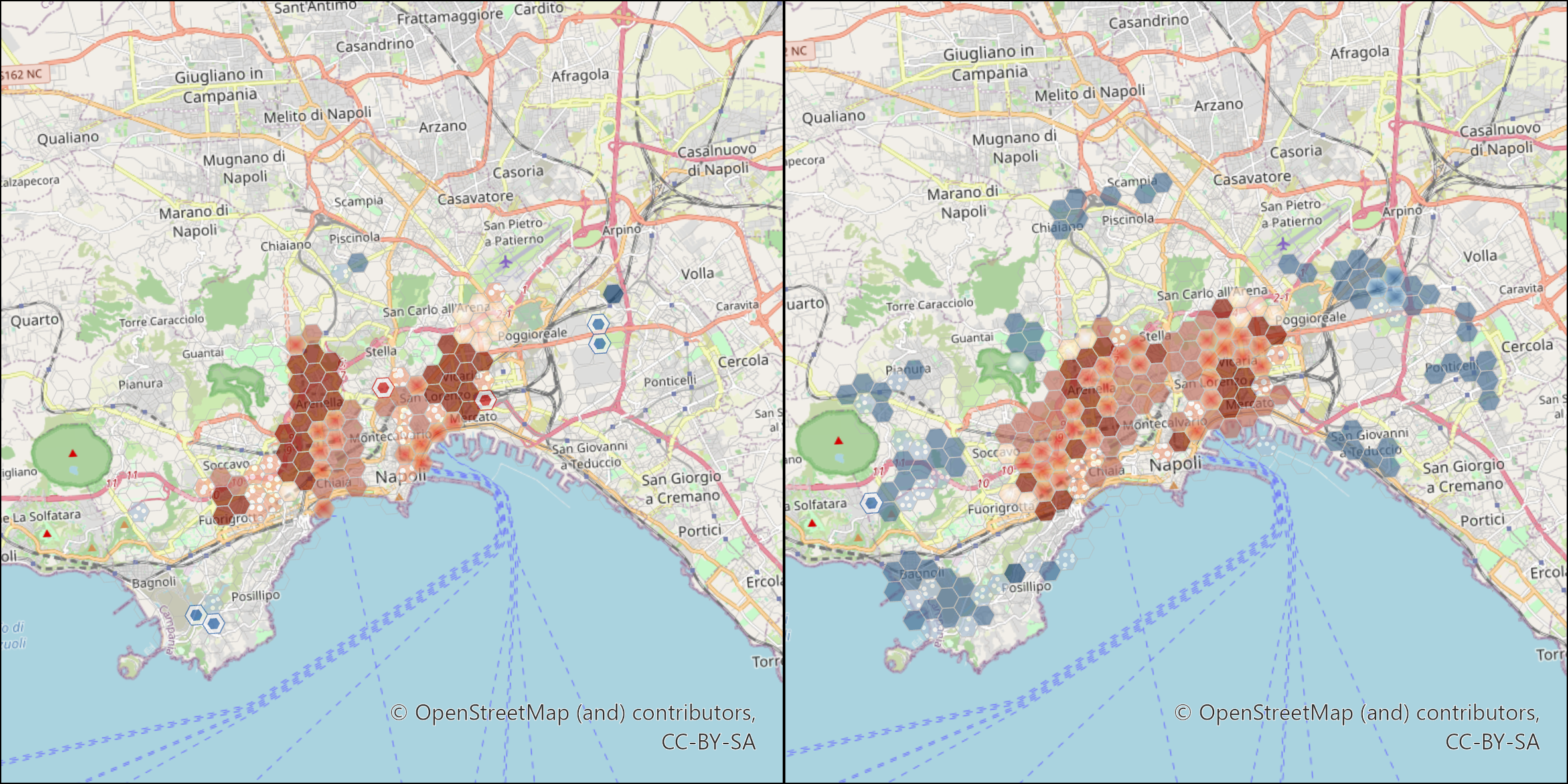

Supplement: S1 File — (ZIP) [file pone.0253868.s001.zip › images/Napoli_real_vs_predicted_12 points_500m_HSA_fullExtent.png]

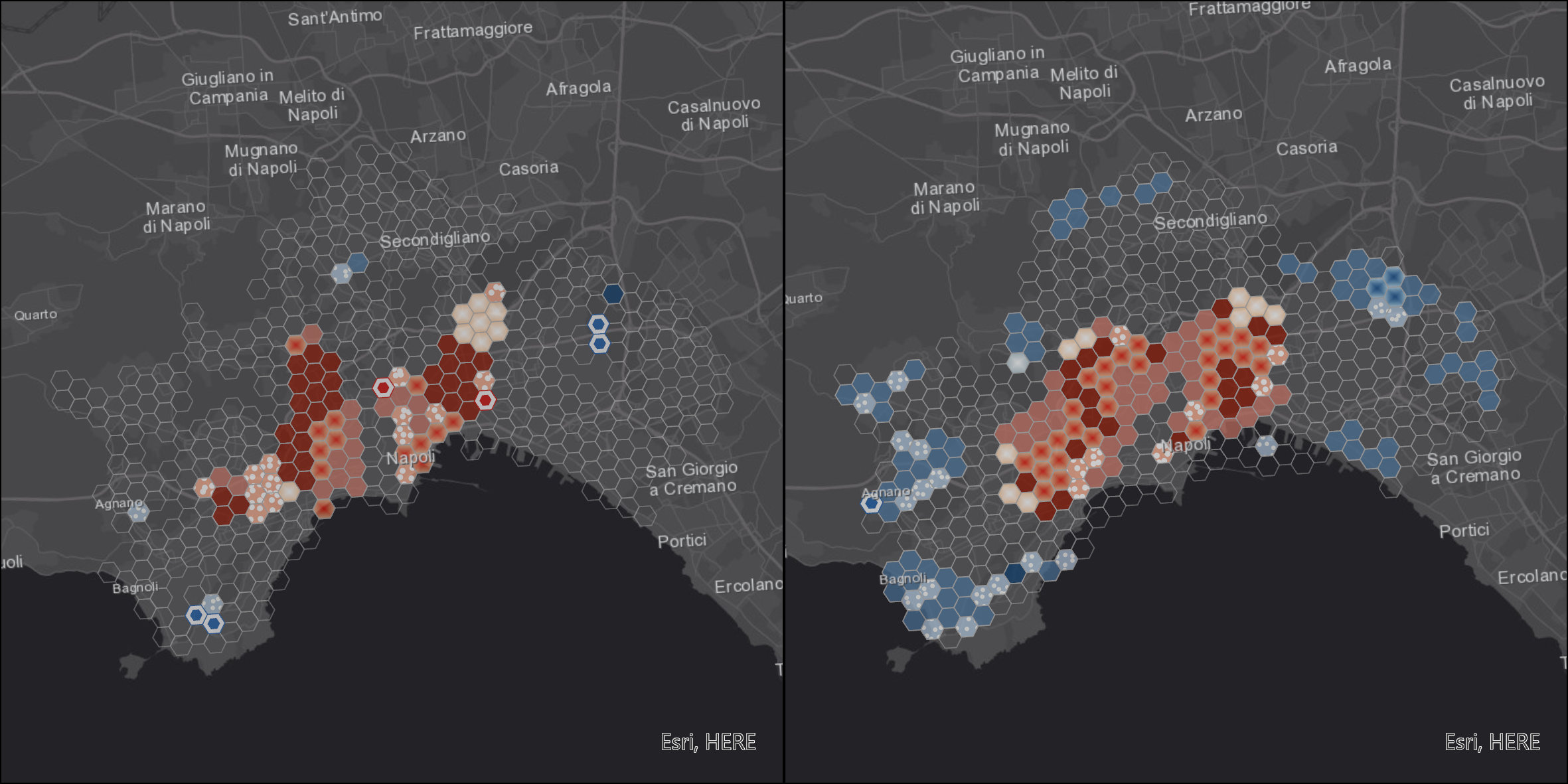

Supplement: S1 File — (ZIP) [file pone.0253868.s001.zip › images/Napoli_real_vs_predicted_12 points_500m_HSA_fullExtent_gray.png]

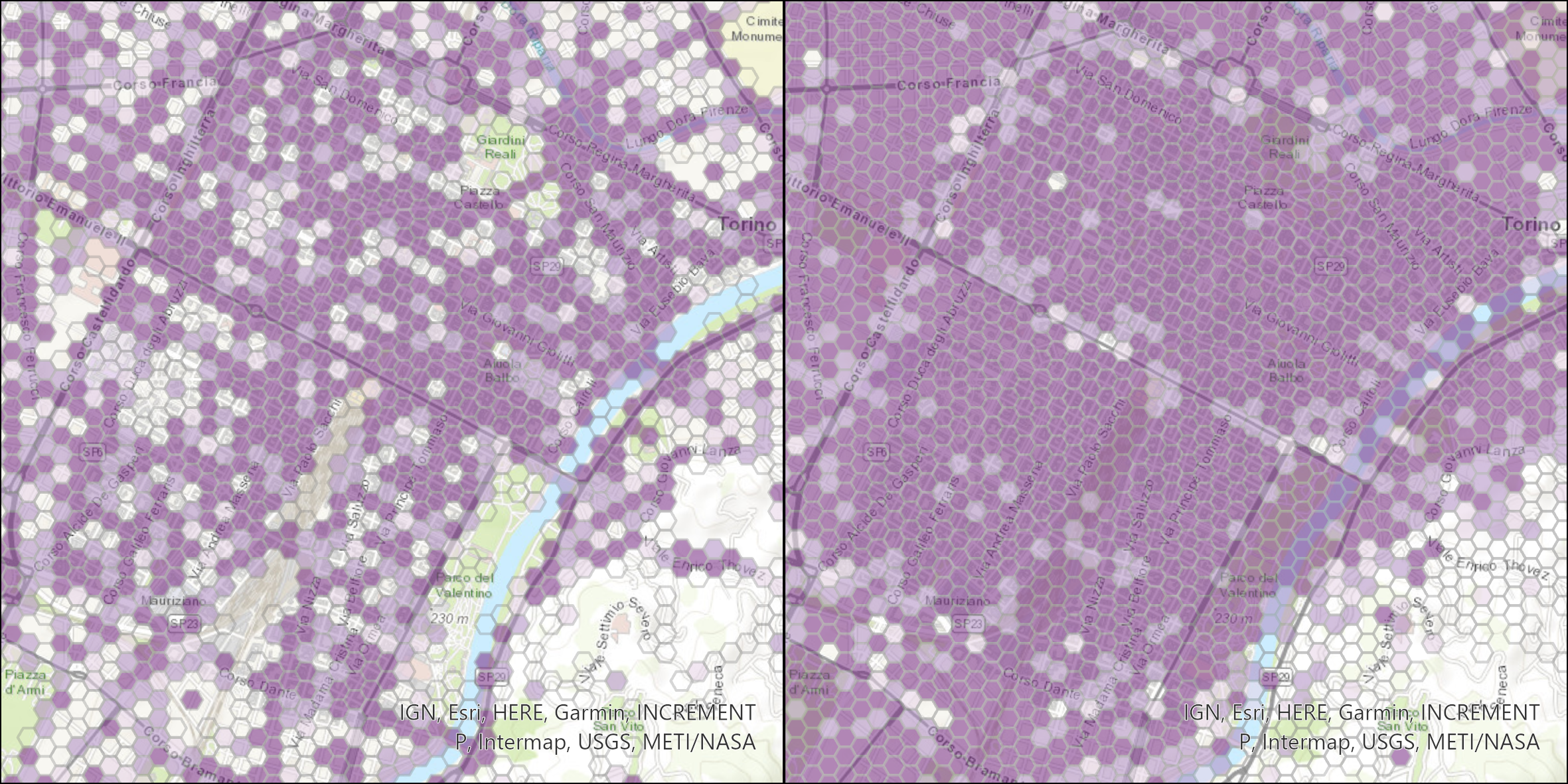

Supplement: S1 File — (ZIP) [file pone.0253868.s001.zip › images/real_vs_predicted_12 points_100m.png]

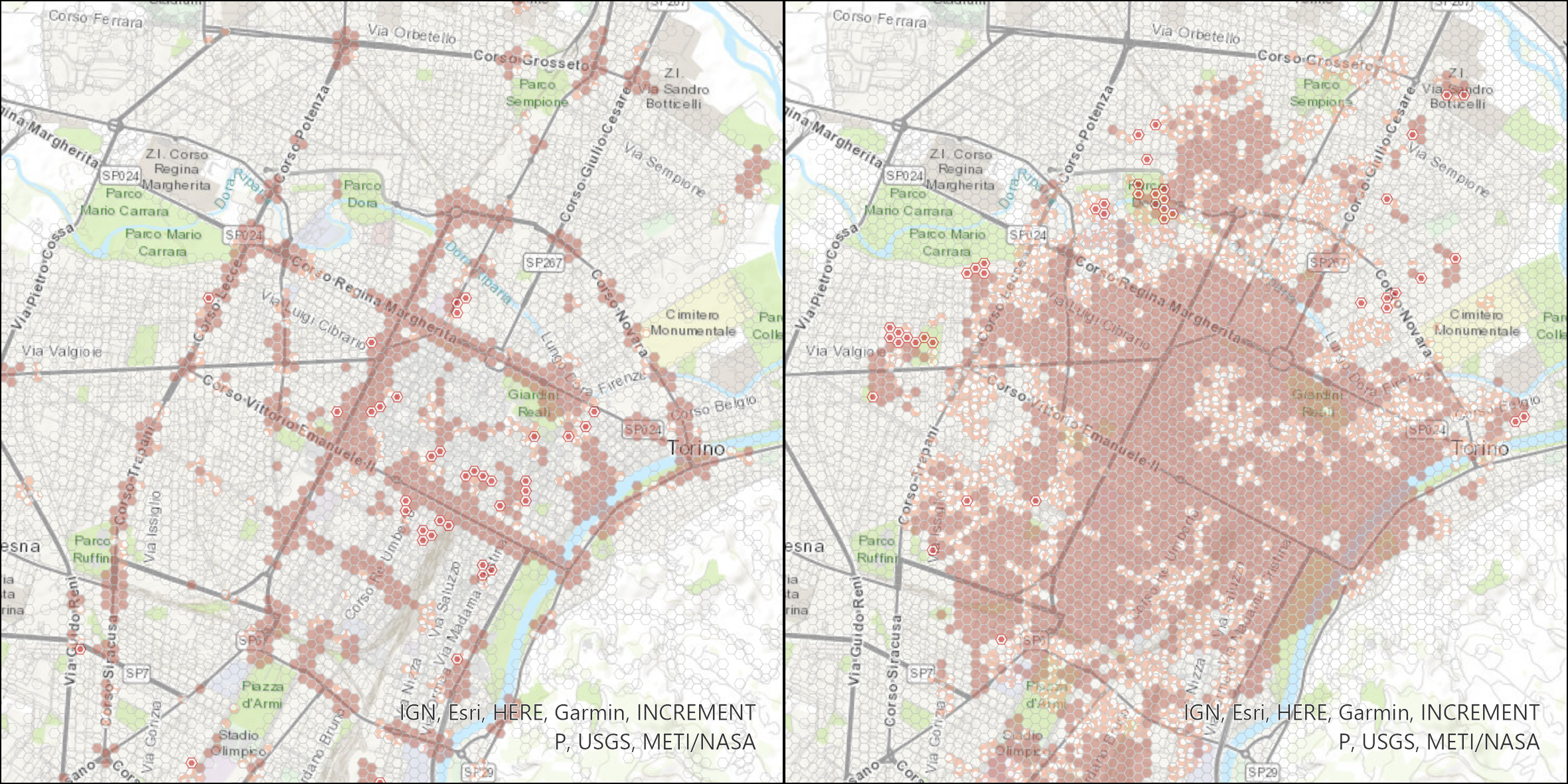

Supplement: S1 File — (ZIP) [file pone.0253868.s001.zip › images/real_vs_predicted_12 points_100m_HSA_midExtent.png]

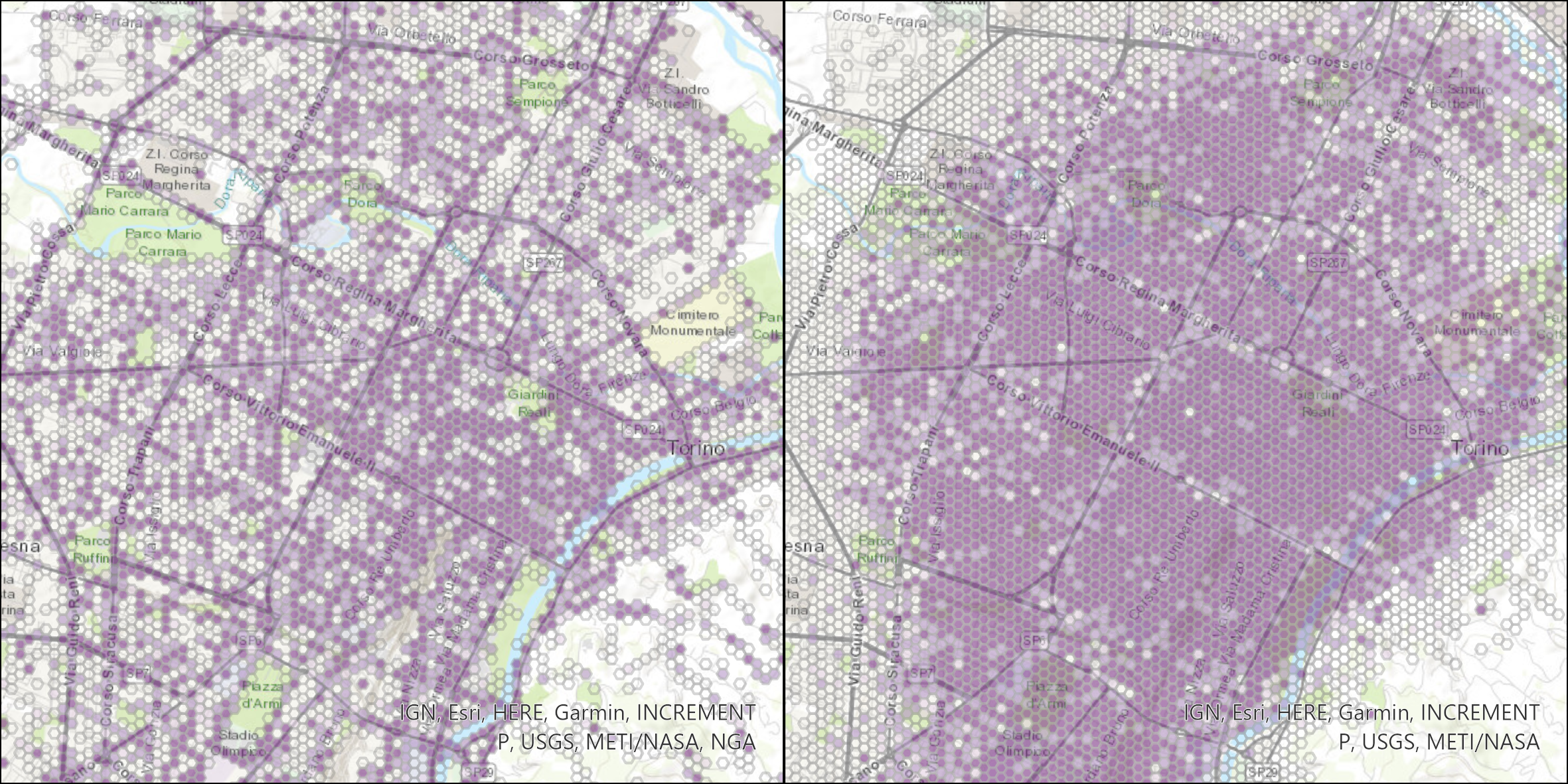

Supplement: S1 File — (ZIP) [file pone.0253868.s001.zip › images/real_vs_predicted_12 points_100m_midExtent.png]

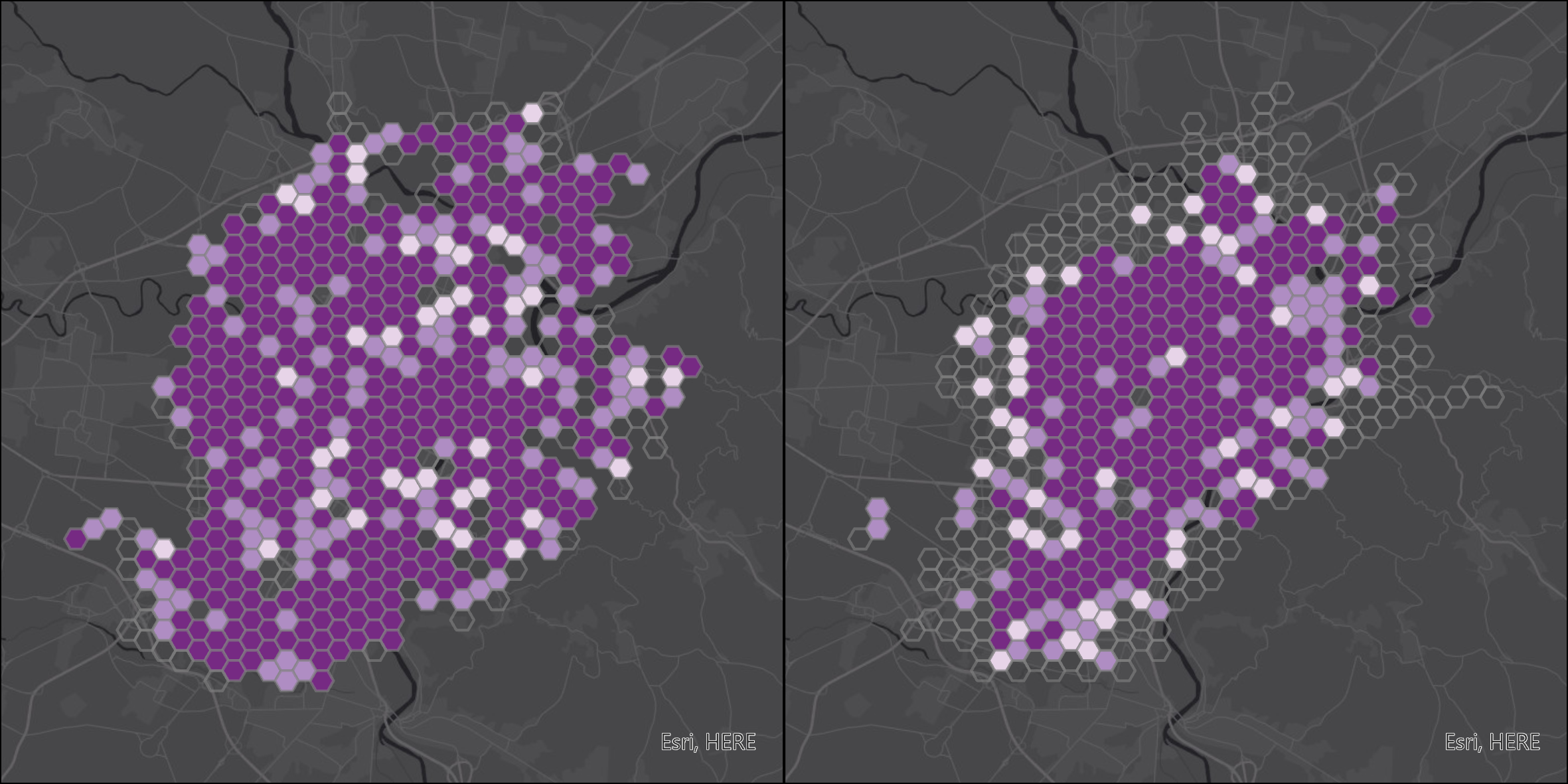

Supplement: S1 File — (ZIP) [file pone.0253868.s001.zip › images/real_vs_predicted_12 points_500m.png]

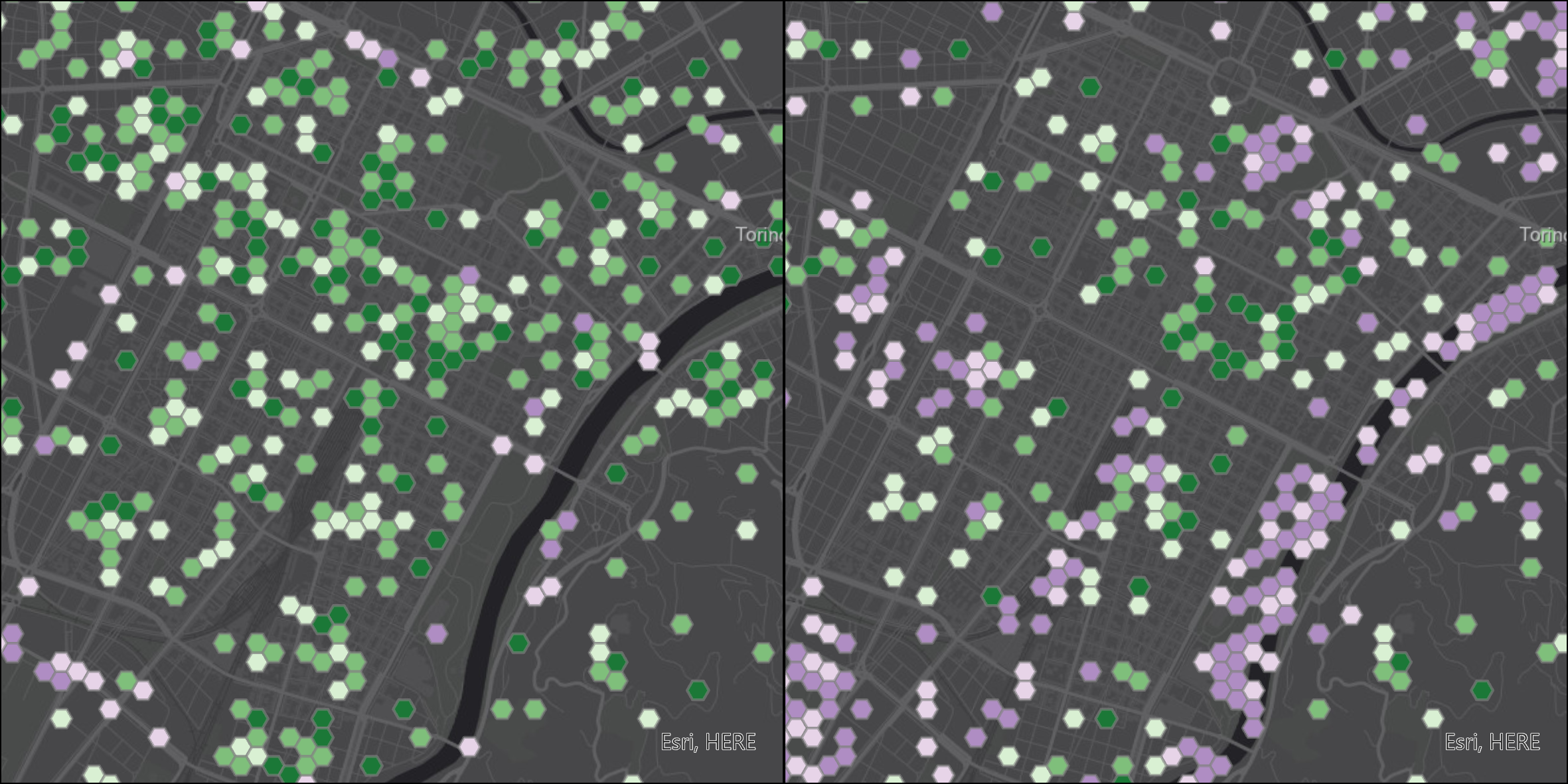

Supplement: S1 File — (ZIP) [file pone.0253868.s001.zip › images/real_vs_predicted_4 points_100m.png]

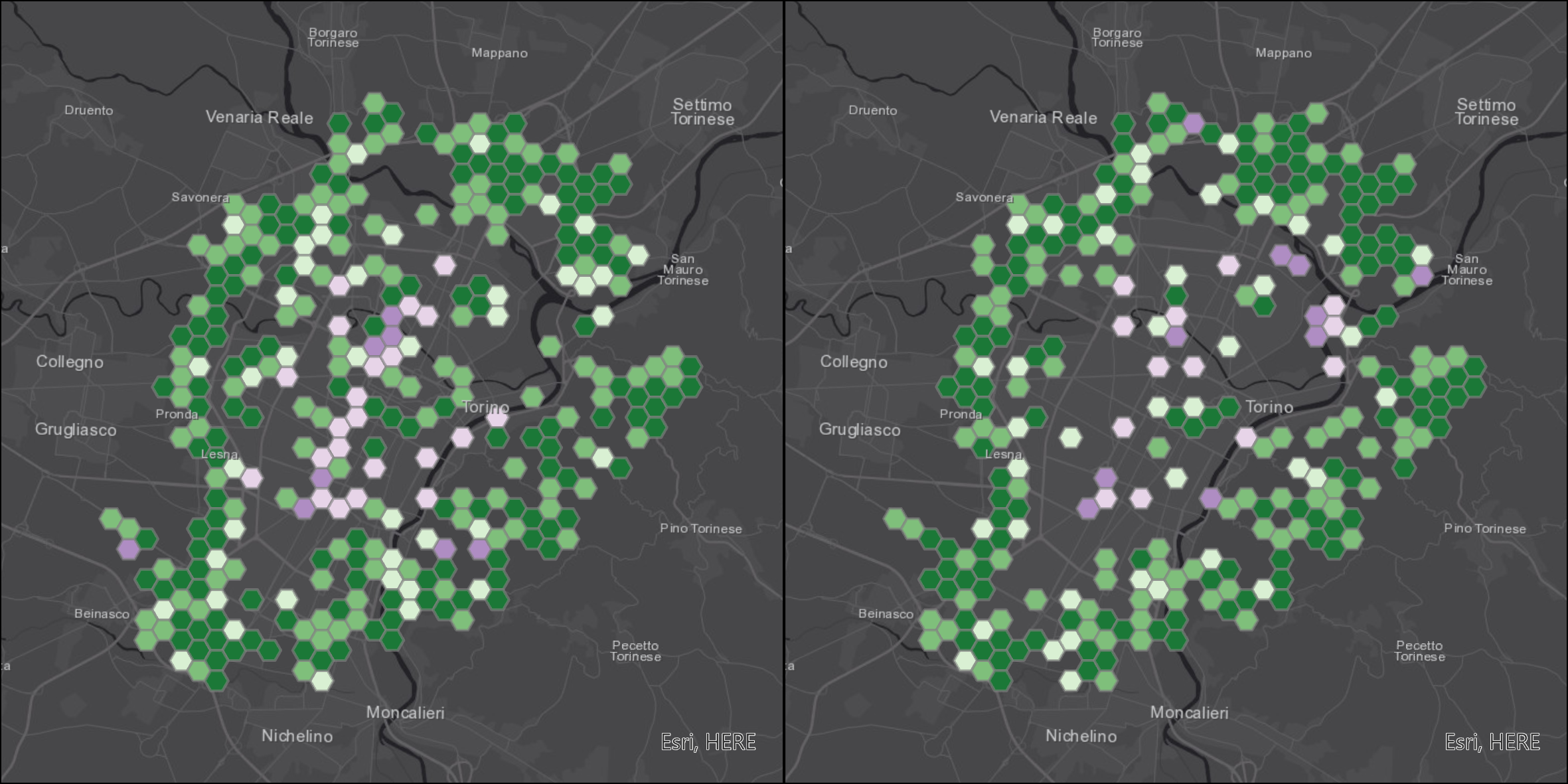

Supplement: S1 File — (ZIP) [file pone.0253868.s001.zip › images/real_vs_predicted_4 points_500m.png]

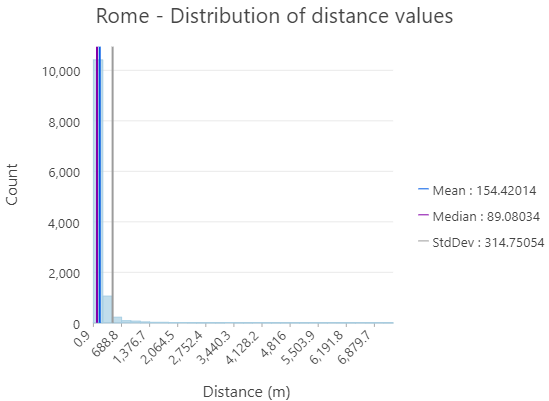

Supplement: S1 File — (ZIP) [file pone.0253868.s001.zip › images/Roma_NearTable_Dist.png]

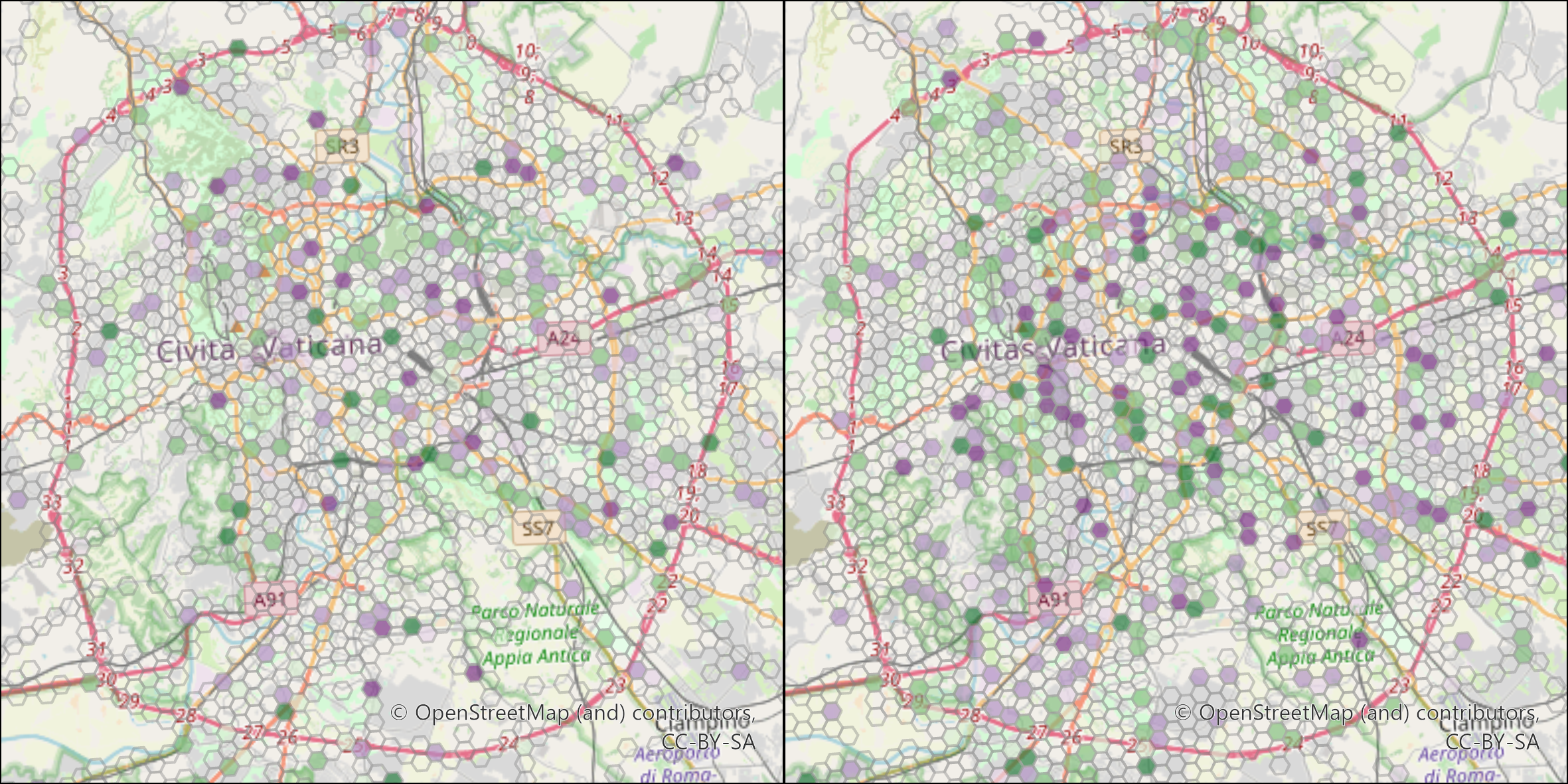

Supplement: S1 File — (ZIP) [file pone.0253868.s001.zip › images/Roma_real_vs_predicted_12 points_500m_fullExtent.png]

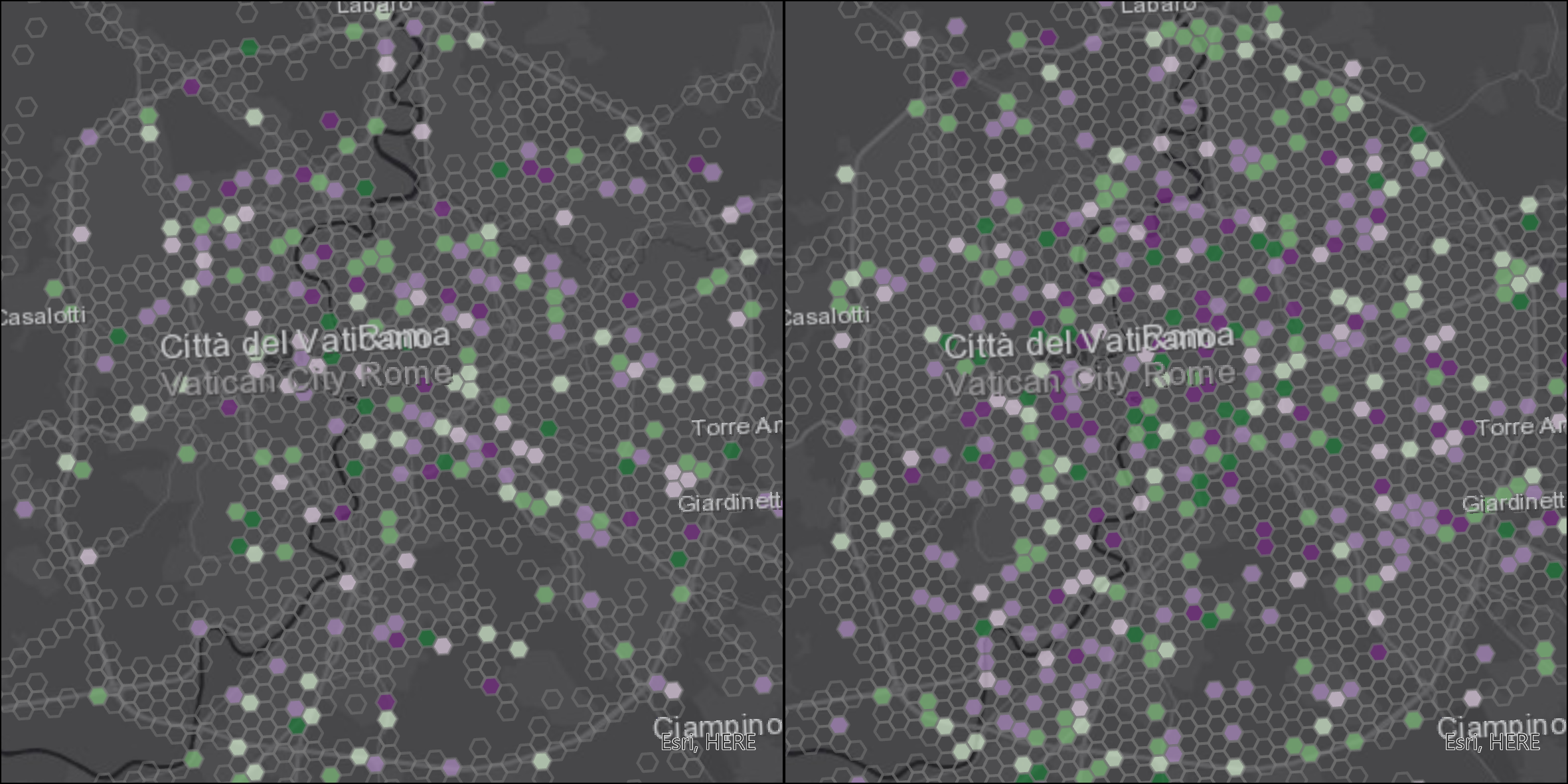

Supplement: S1 File — (ZIP) [file pone.0253868.s001.zip › images/Roma_real_vs_predicted_12 points_500m_fullExtent_gray.png]

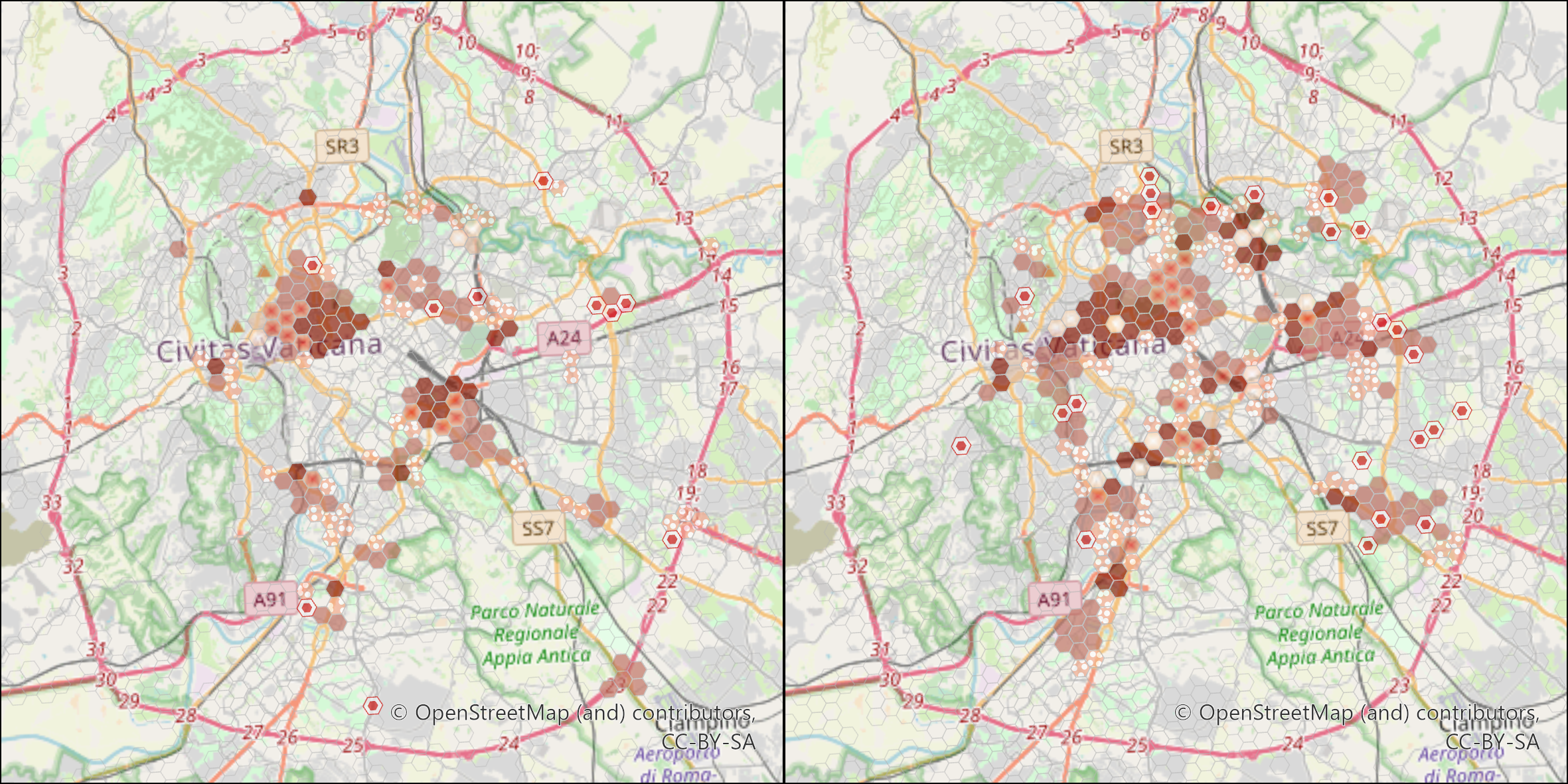

Supplement: S1 File — (ZIP) [file pone.0253868.s001.zip › images/Roma_real_vs_predicted_12 points_500m_HSA_fullExtent.png]

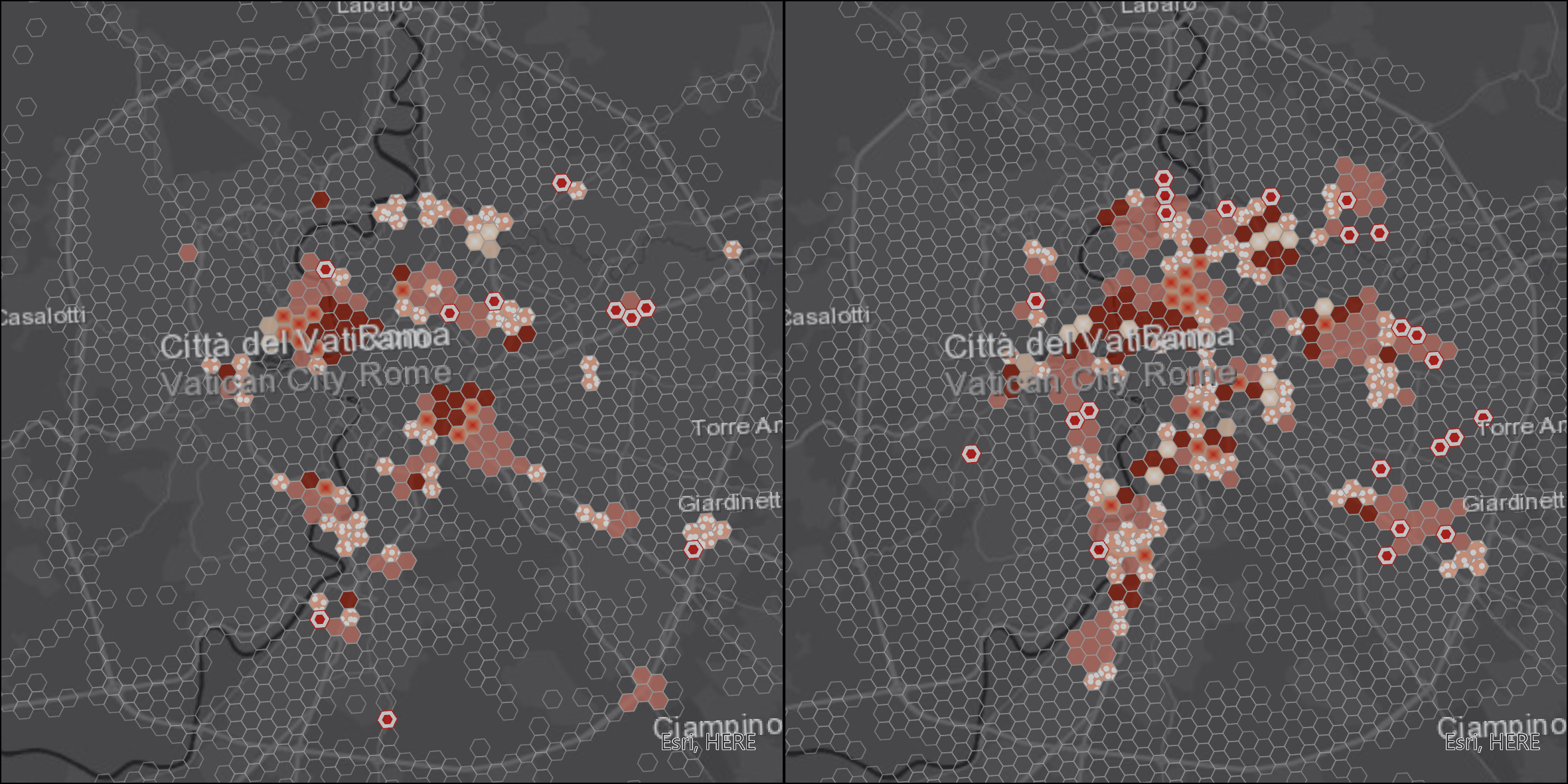

Supplement: S1 File — (ZIP) [file pone.0253868.s001.zip › images/Roma_real_vs_predicted_12 points_500m_HSA_fullExtent_gray.png]

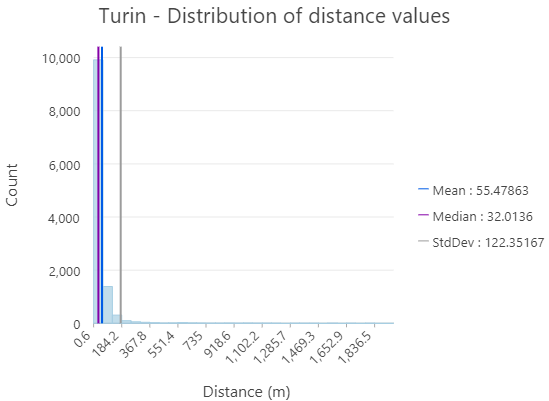

Supplement: S1 File — (ZIP) [file pone.0253868.s001.zip › images/Torino_NearTable_Dist.png]

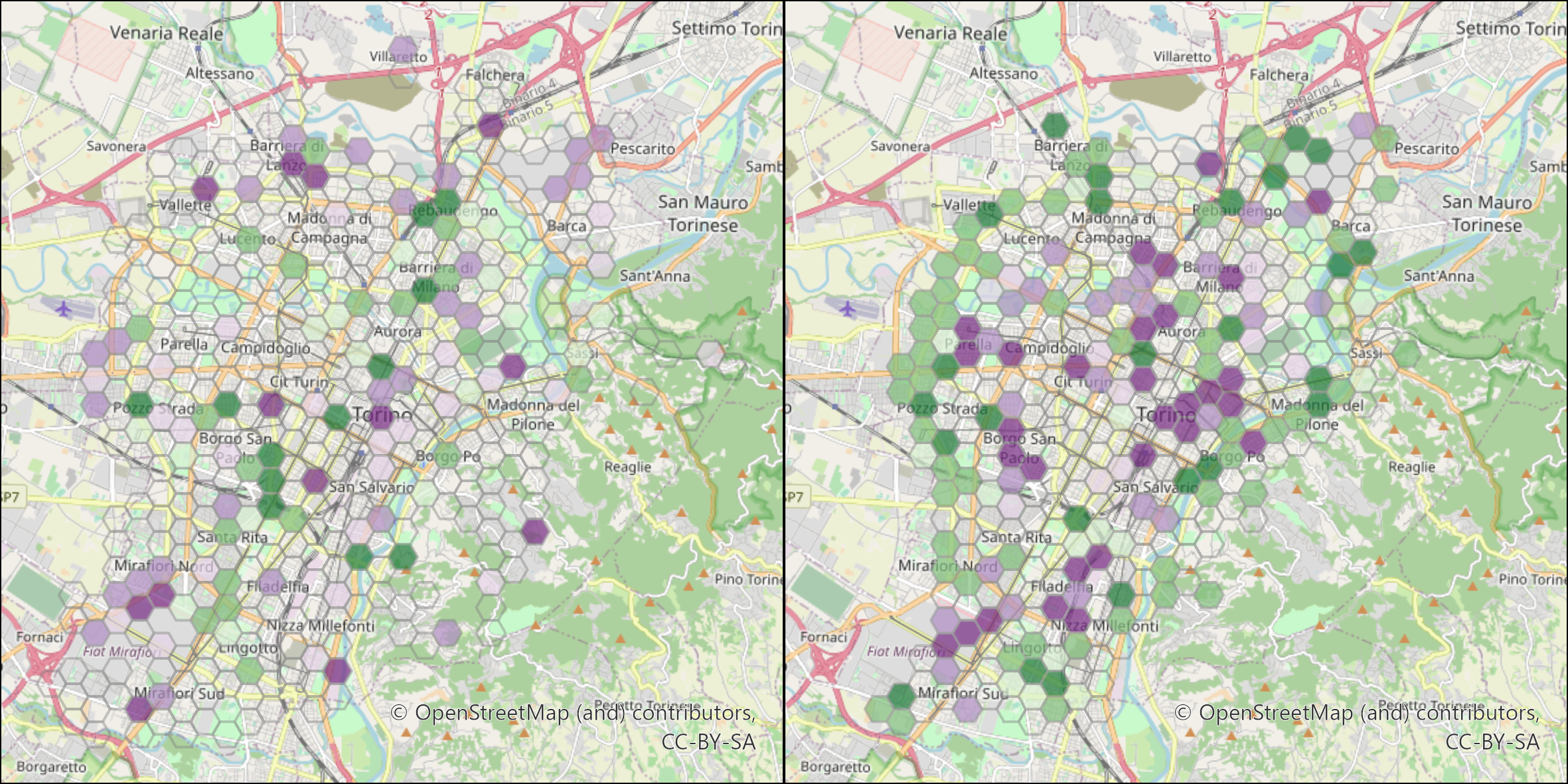

Supplement: S1 File — (ZIP) [file pone.0253868.s001.zip › images/Torino_real_vs_predicted_12 points_500m_fullExtent.png]

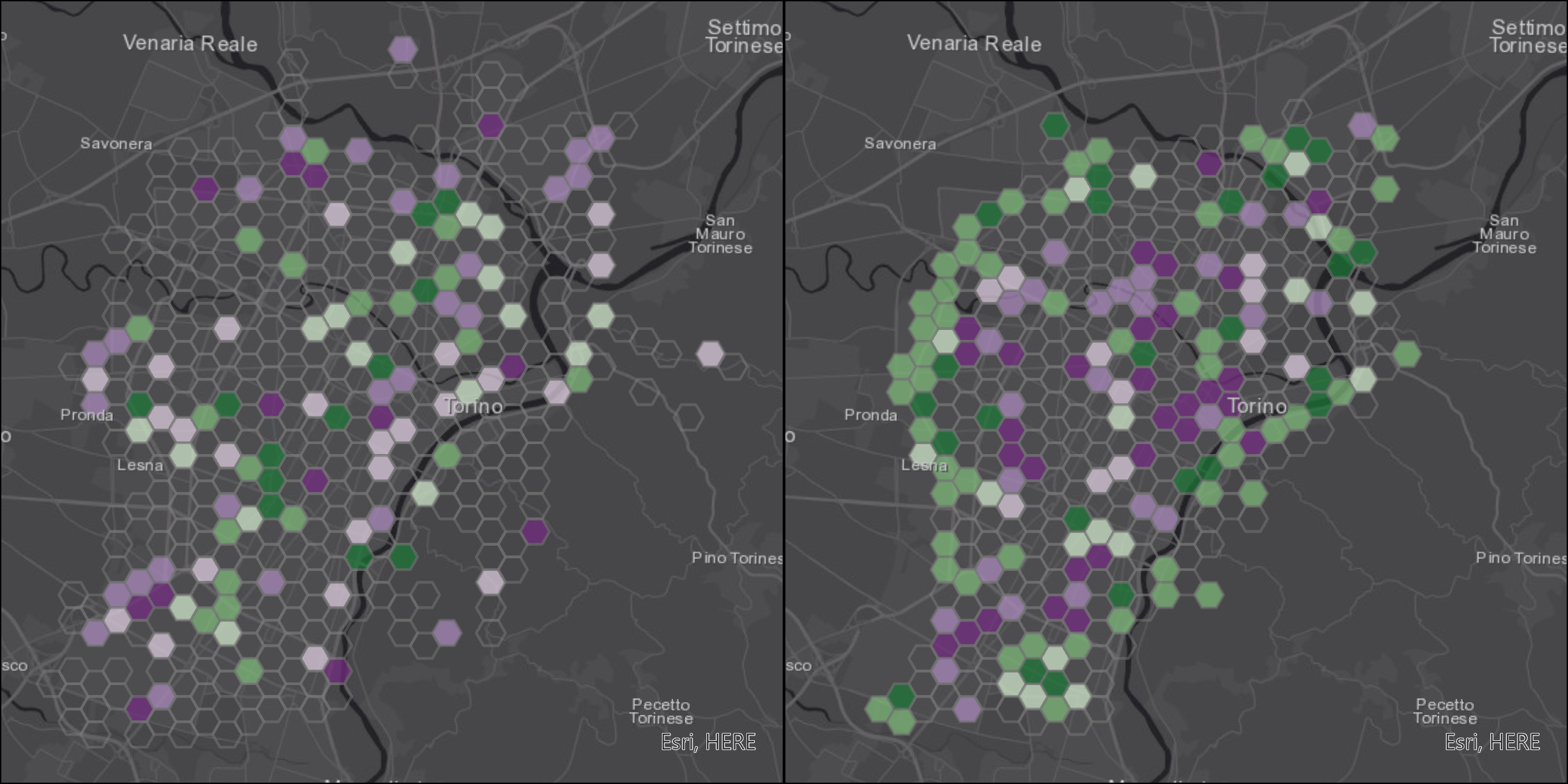

Supplement: S1 File — (ZIP) [file pone.0253868.s001.zip › images/Torino_real_vs_predicted_12 points_500m_fullExtent_gray.png]

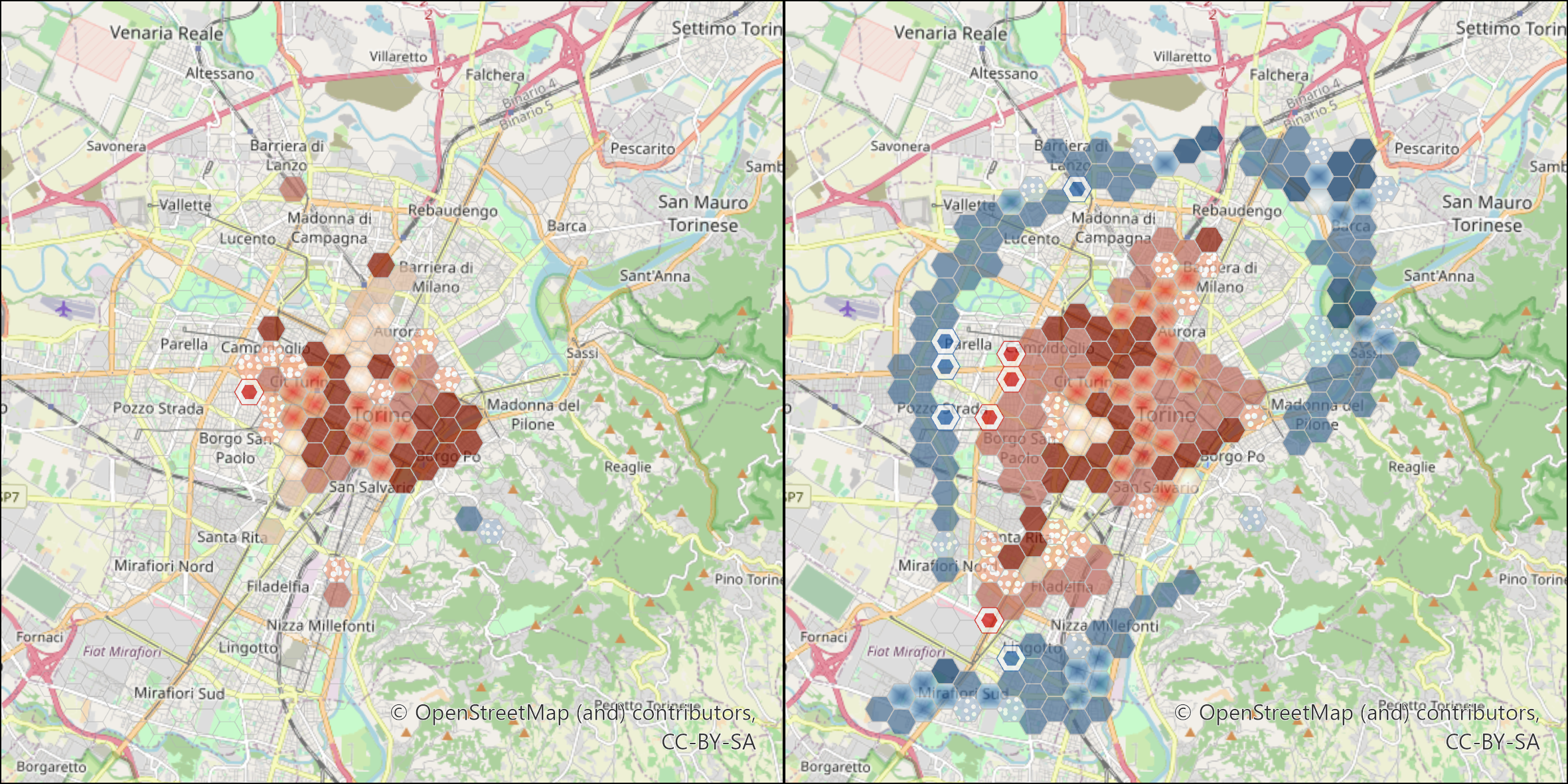

Supplement: S1 File — (ZIP) [file pone.0253868.s001.zip › images/Torino_real_vs_predicted_12 points_500m_HSA_fullExtent.png]

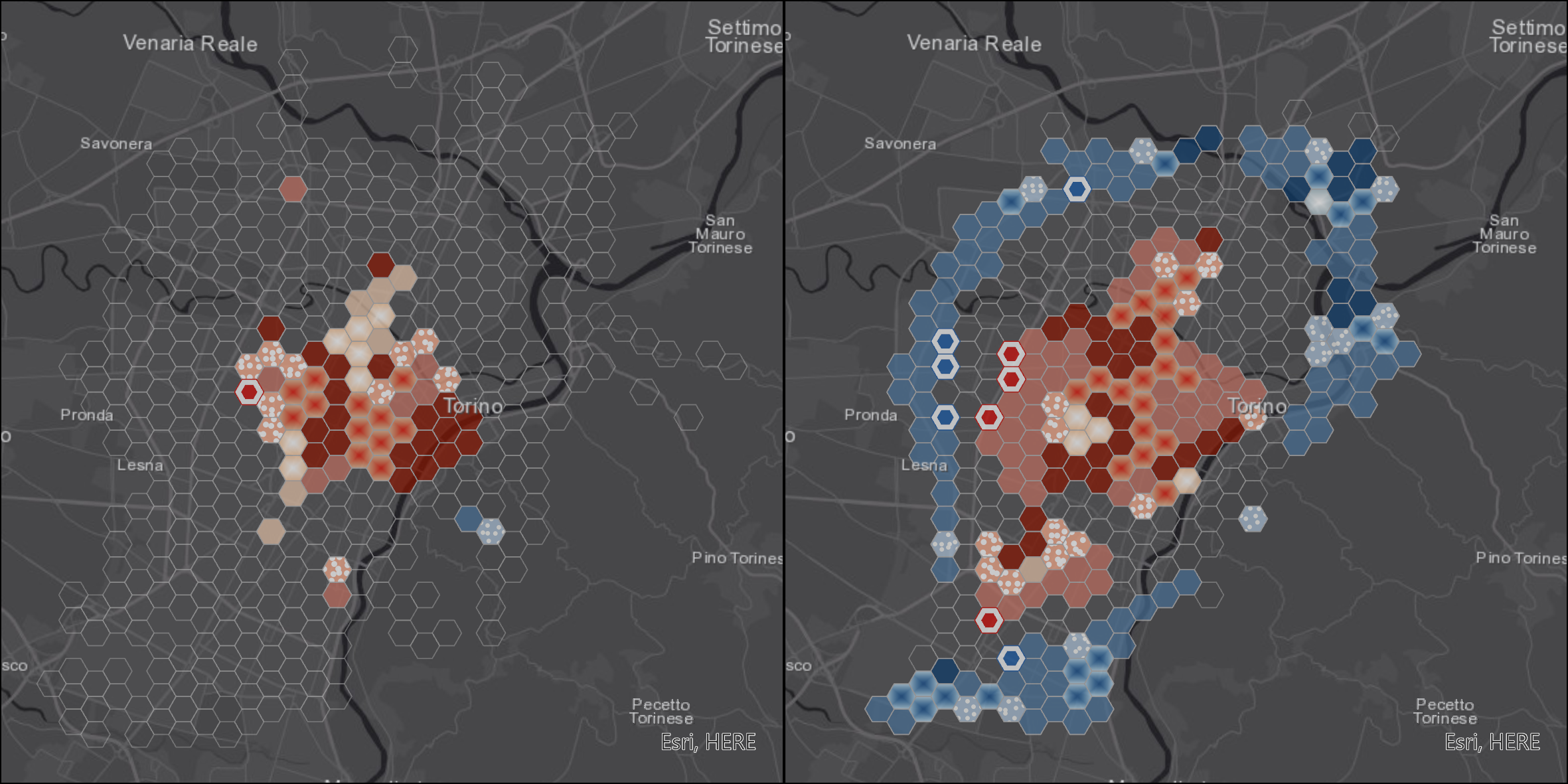

Supplement: S1 File — (ZIP) [file pone.0253868.s001.zip › images/Torino_real_vs_predicted_12 points_500m_HSA_fullExtent_gray.png]

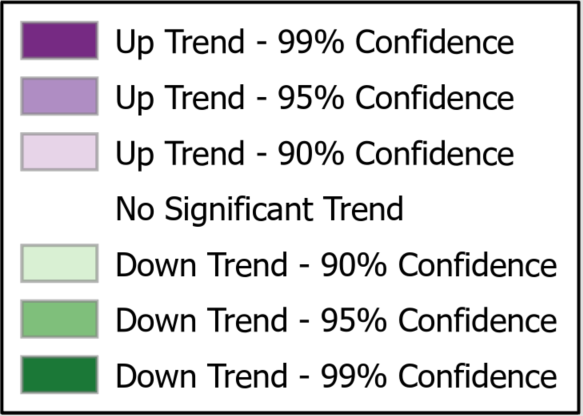

Supplement: S1 File — (ZIP) [file pone.0253868.s001.zip › images/Trends_legend.png]
